# Overexpression of zinc finger protein 687 enhances tumorigenic capability and promotes recurrence of hepatocellular carcinoma

**DOI:** 10.1038/oncsis.2017.63

**Published:** 2017-07-24

**Authors:** T Zhang, Y Huang, W Liu, W Meng, H Zhao, Q Yang, S-J Gu, C-C Xiao, C-C Jia, B Zhang, Y Zou, H-P Li, B-S Fu

**Affiliations:** 1 Department of Hepatic Surgery and Liver transplantation Center of the Third Affiliated Hospital, Organ Transplantation Institute, Sun Yat-sen University, Organ Transplantation Research Center of Guangdong Province, Guangzhou, China; 2 Department of Thyroid and Breast Surgery, The Third Affiliated Hospital of Sun Yat-sen University, Guangzhou, China; 3 Guangdong Key Laboratory of Liver Disease Research, The Third Affiliated Hospital of Sun Yat-sen University, Guangzhou, China; 4 Department of Medical Imaging, The First Affiliated Hospital of Sun Yat-sen University, Guangzhou, China; 5 Department of Medical Oncology, The First Affiliated Hospital of Sun Yat-sen University, Guangzhou, China

## Abstract

Zinc finger protein 687 (ZNF687), identified as a C2H2 zinc finger protein, has been found to be mutated and upregulated in giant cell tumor of bone and acute myeloid leukemia, suggesting an oncogenic role for ZNF687 in cancer. However, the clinical significance and precise role of ZNF687 in cancer progression are largely unknown. Herein, we report that ZNF687 was markedly upregulated in hepatocellular carcinoma (HCC) cell lines and HCC tissues, and was significantly correlated with relapse-free survival in HCC. ZNF687 overexpression greatly enhanced HCC cell capability for tumorsphere formation, invasion and chemoresistance *in vitro*, whereas inhibiting ZNF687 reduced these capabilities and inhibited HCC cell tumorigenic capability *in vivo*. Importantly, extreme limiting dilution analysis revealed that even 1 × 10^2^ ZNF687-transduced cells could form tumors *in vivo*, indicating that ZNF687 contributes to HCC recurrence. Moreover, we demonstrate that ZNF687 transcriptionally upregulated the expression of the pluripotency-associated factors *BMI1*, *OCT4* and *NANOG* by directly targeting their promoters. Therefore, our results suggest that ZNF687 has a promoter role in regulating HCC progression, which provides a potential therapeutic target for HCC in humans.

## Introduction

Hepatocellular carcinoma (HCC), one of the most malignant human cancers, is the third most common cause of cancer-related death globally.^1^ Approximately 700 000 new cases are diagnosed each year worldwide; the estimated deaths caused by liver cancer around the world exceed 600 000.^2^ More than 50% of new cases and deaths occur in China.^3^ Despite the rapid development of clinical treatment, such as liver transplantation, surgical resection, and chemotherapy, the long-term prognosis for patients with HCC remains unsatisfactory due to the high rate of recurrence.^4^ Therefore, identifying effective therapeutic strategies and the molecular mechanisms underlying HCC recurrence would be of great clinical value.

Cancer stem cells (CSCs), a small subpopulation of cancer cells, are capable of self-renewal and have multi-lineage differentiation potential, which results in metastasis, chemotherapy resistance, and recurrence in many cancers, including HCC.^5, 6, 7^ For example, the CD133^+^ and CD44^+^ CSC subpopulation in HCC can form tumor spheroids *in vitro* and promote tumorigenic capability *in vivo*;^7, 8, 9^ the CD24^+^ CSC subpopulation in HCC has self-renewal and tumor initiation capability, and confers resistance to cisplatin via STAT3 (signal transducer and activator of transcription 3)-mediated NANOG (Nanog homeobox) regulation.^10^ Furthermore, the presence of CSCs is associated with worse overall survival and higher recurrence rates, suggesting that CSCs might be an attractive therapeutic target in HCC.^7, 10^ Importantly, eliminating CSCs in pancreatic cancer, breast cancer and leukemia with therapeutic agents prevents cancer progression.^11, 12, 13^ Therefore, targeting CSCs might improve the cure rates and survival of patients with HCC.

Multiple pluripotency-associated factors are overexpressed in CSCs and are associated with cancer progression, chemoresistance, and recurrence.^14, 15^ For example, BMI1 (BMI proto-oncogene, polycomb ring finger), a major component of the polycomb group complex 1 (PRC1), is essential for self-renewal in both normal cells and CSCs.^16^ *BMI1* overexpression promotes HCC cell proliferation and invasion *in vitro* and induces the formation of poorly differentiated tumors *in vivo*, suggesting that it might represent a potential molecular target of HCC.^16^ The DNA-binding homeobox transcription factors NANOG and OCT4 (POU domain class 5, transcription factor 1) are required for maintaining stem cell pluripotency.^17, 18^ Furthermore, *NANOG* and *OCT4* overexpression regulates HCC stem cell self-renewal, induces resistance to therapeutic agents, and promotes tumorigenic capability,^19, 20^ which correlates with worse clinical outcome. Hence, investigating the mechanisms and regulatory pathways of CSC self-renewal and pluripotency is essential for treating cancer.

Zinc finger protein 687 (*ZNF687*) is a newly identified C2H2 zinc finger factor. *ZNF687* mutations have been observed in severe Paget disease of bone associated with tumor tissue of giant cell tumor (GCT),^21^ and *ZNF687* is translocated with *RUNX1* (runt-related transcription factor 1) in acute myeloid leukemia (AML),^22^ which suggests that *ZNF687* might be a potent oncogene. In the present study, we found that *ZNF687* expression correlated with poor overall survival and relapse-free survival in HCC. *ZNF687* overexpression greatly promoted HCC stem cell-like traits and improved tumorigenic capability via transcriptional upregulation of the pluripotency-associated factors *BMI1*, *OCT4* and *NANOG*. These results indicate that *ZNF687* potentially has a critical oncogenic role in HCC progression and might represent a novel, valuable therapeutic target in HCC.

## Results

### *ZNF687* overexpression correlated with poor prognosis in HCC

**Figure 1:**
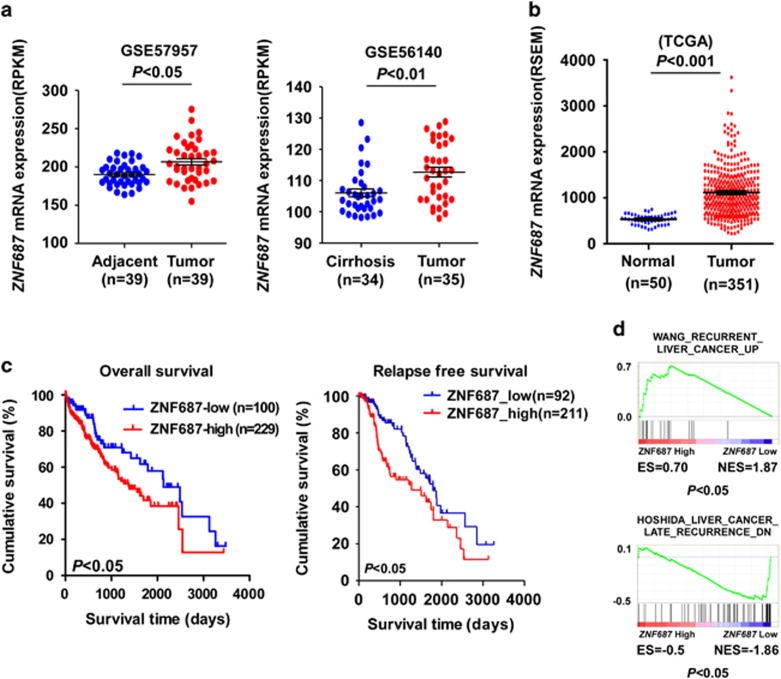
*ZNF687* overexpression correlates with HCC progression and recurrence. (**a**) *ZNF687* mRNA expression profile in HCC tissues and adjacent cancer tissues and in cirrhosis liver tissue from the GSE57957 (*P*<0.05) and GSE56140 data sets (*P*<0.01). (**b**) *ZNF687* mRNA expression profile in HCC tissues (*n*=351) and normal liver tissues (*n*=50) from TCGA data set (*P*<0.001). (**c**) Kaplan–Meier analysis of overall survival curves and relapse-free survival curves from TCGA data set for patients with HCC with relatively low or relatively high ZNF687 expression levels (*P*<0.05). (**d**) GSEA plot showing that high *ZNF687* expression correlated positively with recurrence gene signatures in published TCGA expression profiles of patients with HCC.

*ZNF687* mutations or translocation is closely correlated with cancer development and progression.^21, 22^ Analysis of *ZNF687* status in The Cancer Genome Atlas (TCGA) data sets did not reveal obvious *ZNF687* mutations or translocation in HCC. However, analysis of publicly available data sets showed that *ZNF687* mRNA expression was significantly upregulated in HCC tissues compared with adjacent tumor tissues or cirrhosis liver tissues (GSE57957, GSE56140 and TCGA) (Figures 1a and b). Kaplan–Meier analysis of TCGA data sets revealed that *ZNF687* mRNA expression was significantly correlated with poor overall survival and relapse-free survival in HCC (*P*<0.05; *P*<0.05, respectively; Figure 1c), indicating that *ZNF687* might be involved in HCC progression. Moreover, gene set enrichment analysis (GSEA) of TCGA data set revealed that *ZNF687* gene expression correlated positively with the recurrence-related gene signatures (Figure 1d), suggesting that *ZNF687* upregulation may contribute to HCC recurrence.

**Figure 2:**
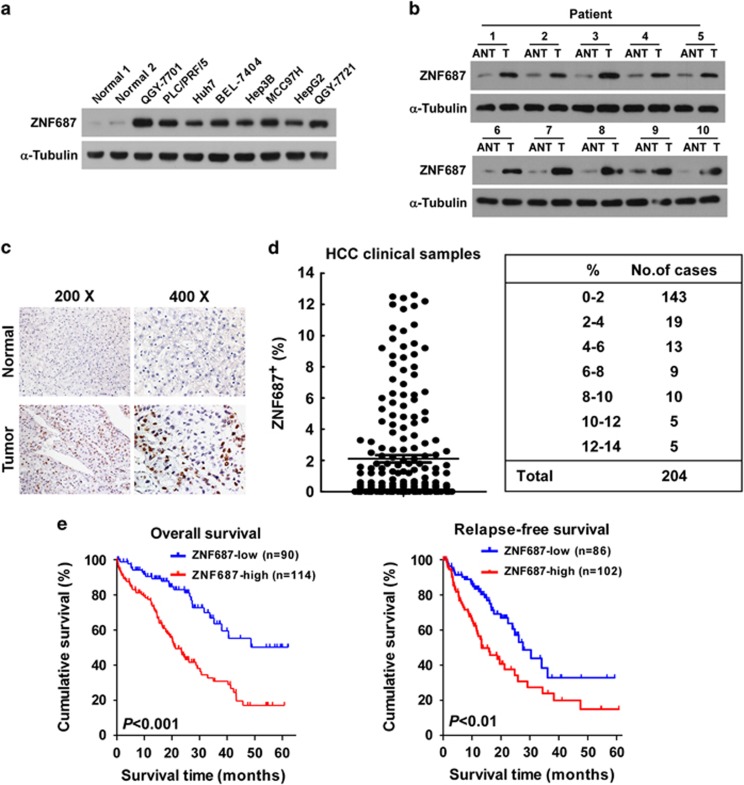
*ZNF687* overexpression correlates with poor prognosis of HCC. (**a**, **b**) Western blot analysis of ZNF687 expression in two primary normal human liver cell lines and eight HCC cell lines (**a**) and in ten paired primary HCC tissues (T) and the matched adjacent non-tumor tissues (ANT) from the same patient (**b**). α-Tubulin, loading control. (**c**) IHC staining showing upregulated ZNF687 protein expression in HCC specimens compared with normal liver tissues. (**d**) IHC statistical analysis showing that 70% of HCC clinical specimens had <2% ZNF687 expression. Error bars, mean±s.d. of three independent experiments. (**e**) Kaplan–Meier curves of overall survival (left) and relapse-free survival (right) in patients with HCC with relatively low-ZNF687 expression versus relatively high ZNF687 expression (*n*=204; *P*<0.01, log-rank test).

To validate the above analyses, we detected ZNF687 mRNA and protein expression in HCC cell lines and clinical HCC tissues. Consistent with TCGA analysis, the expression of both ZNF687 mRNA and protein was markedly higher in the eight HCC cell lines and ten HCC tissues than in the two primary normal human liver cell lines and the matched adjacent non-tumor tissues, respectively (Figures 2a and b; Supplementary Figures 1A,B). To further evaluate the correlation between *ZNF687* expression and HCC clinicopathologic features, 204 archived paraffin-embedded HCC specimens and 10 normal liver tissues were analyzed by immunohistochemical (IHC) staining with anti-human ZNF687 antibody (Figure 2c). ZNF687 was overexpressed in HCC tissues (*n*=204) but was undetectable in normal liver tissue (*n*=10), and the HCC specimens contained 0–12.6% ZNF687^+^ cells, whereas there was no ZNF687 expression in the non-tumor tissues, and ZNF687 was expressed in less than 2% of cells in >70% of the HCC cases (143/204) (Figures 2c and d). Statistical analysis of the IHC-stained sections revealed that ZNF687 protein expression was significantly correlated with clinical stage (*P*<0.001), histological differentiation (*P*<0.001), T classification (*P*<0.001), N classification (*P*<0.05) and M classification (*P*<0.05), but no correlations were found with age, gender or hepatitis B surface antigen (HBsAg) and alpha-fetoprotein (AFP) expression level (Supplementary Tables 1 and 2).

Next, we determined whether ZNF687 protein upregulation correlated with poor prognosis as HCC progressed. Kaplan–Meier analysis and log-rank testing revealed that ZNF687 protein expression levels in the HCC specimens were inversely correlated with overall survival (*P*<0.001) and relapse-free survival time (*P*<0.01; Figure 2e), suggesting that ZNF687 contributes to HCC recurrence. Moreover, univariate and multivariate survival analyses indicated that ZNF687 expression was an independent prognostic factor of HCC (Supplementary Table 3). Taken together, our findings suggest that ZNF687 upregulation might contribute to HCC progression and recurrence and represent a prognostic factor in HCC outcome.

### ZNF687 upregulation promoted stem cell-like traits in HCC cells *in vitro*

**Figure 3:**
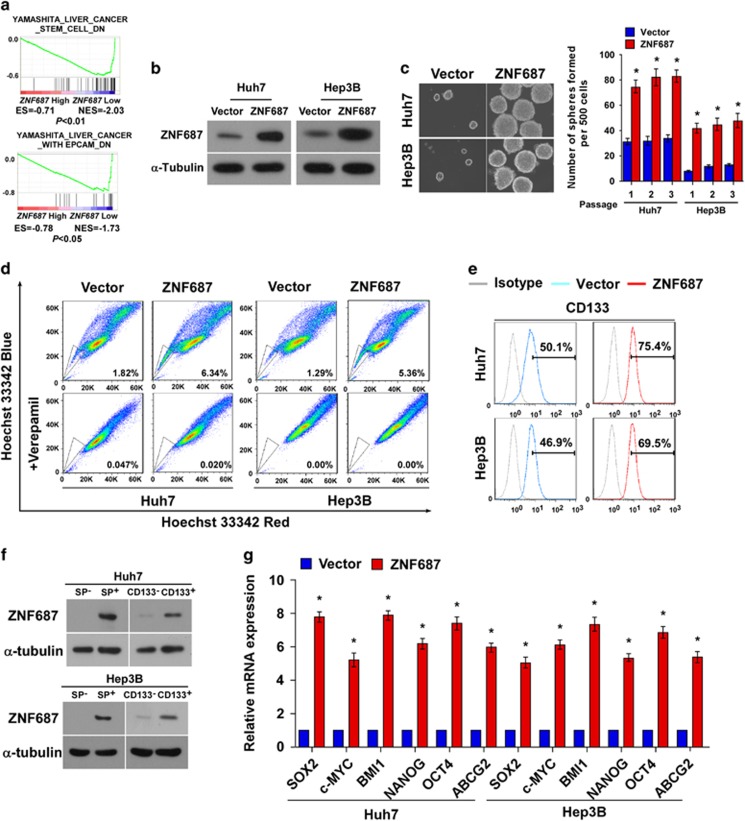
*ZNF687* upregulation promotes HCC CSC-like traits *in vitro*. (**a**) GSEA plot showing that high *ZNF687* expression correlated positively with stem cell gene signatures in published TCGA expression profiles of patients with HCC. (**b**) Western blot analysis of ZNF687 expression in Huh7 and Hep3B cells stably overexpressing ZNF687. (**c**) Representative micrographs of tumorspheres formed. Scale bar, 100 μm. Histogram shows the mean number of tumorspheres formed. (**d**) Hoechst 33342 dye exclusion assay showing that *ZNF687* overexpression promoted the SP^+^ subpopulation. (**e**) Flow cytometry analysis of the CD133^+^ population in Huh7 and Hep3B cells stably overexpressing ZNF687. (**f**) Western blot analysis of ZNF687 expression in SP^+^ or SP^−^ cells and CD133^+^ or CD133^−^ cells. (**g**) Real-time PCR of mRNA expression of the pluripotency-associated factors. Transcript levels were normalized to glyceraldehyde-3-phosphate dehydrogenase (*GAPDH*) expression. Error bars, mean±s.d. of three independent experiments. *P<0.05.

CSCs are involved in regulating HCC recurrence.^5, 6, 7^ Interestingly, GSEA revealed that *ZNF687* gene expression significantly correlated with the gene signatures in liver CSCs, liver cancer with EPCAM (epithelial cell adhesion molecule), hematopoietic stem cells, and lymphoid stem cells (Figure 3a; Supplementary Figure 2), suggesting that *ZNF687* may have a regulatory role in HCC stemness. Consistently, the tumorsphere formation assay showed that ZNF687-transduced cells formed more tumorspheres with higher cell content compared with the spheres formed by vector control cells (Figures 3b and c), suggesting that ZNF687 overexpression promotes the tumorigenic capability of HCC cells *in vitro*. Furthermore, ZNF687 overexpression greatly increased the proportion of SP^+^ cells, a subpopulation of cells with stem cell-like traits:^23^ from 1.82 to 6.34% in Huh7 cells, and from 1.29 to 5.36% in Hep3B cells (Figure 3d). Moreover, the CD133^+^ subpopulation, widely recognized as a HCC CSC marker,^7^ was greatly increased in ZNF687-transduced Huh7 and Hep3B cells as compared with vector control cells (Figure 3e). Importantly, the SP^+^ or CD133^+^ subpopulations had greatly higher ZNF687 expression compared with SP^−^ or CD133^−^ cells (Figure 3f), further demonstrating the vital role of ZNF687 in regulating the stem cell-like traits in HCC cells. The pluripotency-associated factors, including *SOX2* (SRY box 2), c-*MYC* (MYC proto-oncogene, bHLH transcription factor), *BMI1*, *NANOG*, *OCT4* and *ABCG2* (ATP-binding cassette subfamily G member G), were upregulated in ZNF687-overexpressing HCC cells (Figure 3g). Collectively, these results indicate that ZNF687 upregulation promotes stem cell-like traits in HCC cells *in vitro*.

### Silencing ZNF687 inhibited stem cell-like traits in HCC cells *in vitro*

**Figure 4:**
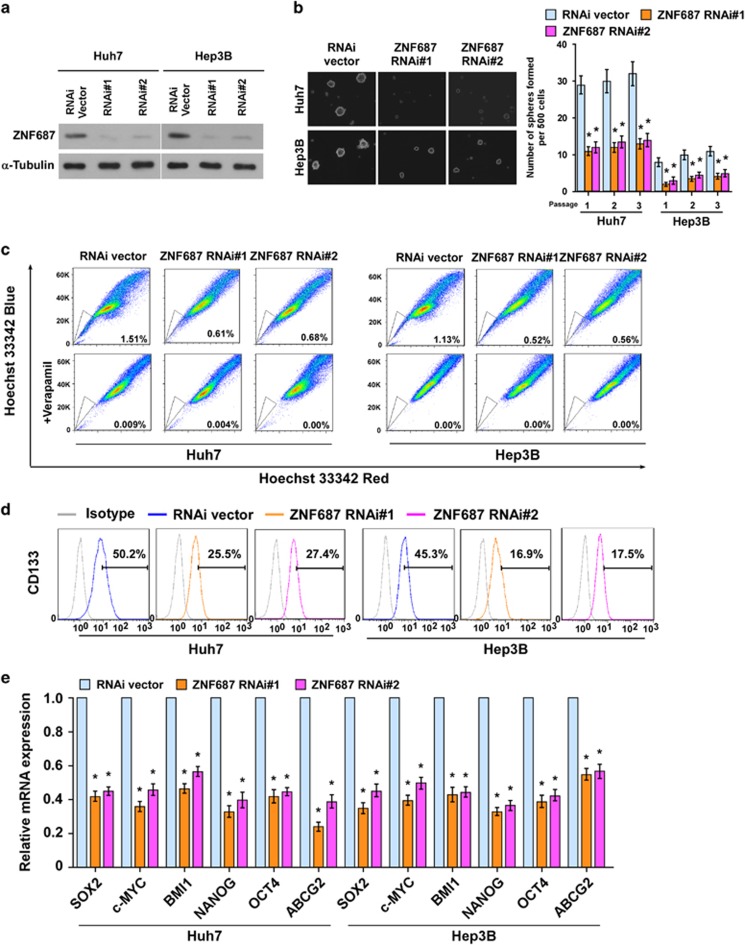
Silencing *ZNF687* inhibits HCC stem cell-like traits *in vitro*. (**a**) Western blot analysis of ZNF687 expression in ZNF687-silenced Huh7 and Hep3B cells. (**b**) Representative micrographs of tumorspheres formed. Scale bar, 100 μm. Histogram shows the mean number of tumorspheres formed. (**c**) Hoechst 33342 dye exclusion assay showing that silencing ZNF687 decreased the SP^+^ subpopulation. (**d**) Flow cytometry analysis of the CD133^+^ population in Huh7 and Hep3B cells stably expressing ZNF687-shRNA(s). (**e**) Real-time PCR of the mRNA expression of the pluripotency-associated factors. Transcript levels were normalized to *GAPDH* expression. Error bars, mean±s.d. of three independent experiments. *P<0.05. RNAi, RNA interference.

We explored the effect of silencing ZNF687 on the stem cell-like traits of HCC cells. Endogenous ZNF687 was silenced using short hairpin RNA (shRNA) (Figure 4a). As expected, silencing ZNF687 decreased tumorsphere number and size (Figure 4b). The proportion of SP^+^ cells (Figure 4c) and CD133^+^ cells (Figure 4d) was decreased in ZNF687-silenced HCC cells as compared with control cells. Furthermore, silencing ZNF687 significantly reduced the mRNA expression of the pluripotency-associated factors, including *SOX2*, c-*MYC*, *BMI1*, *NANOG*, *OCT4* and *ABCG2* (Figure 4e). Altogether, these findings further support the premise that ZNF687 regulates stem cell-like traits in HCC.

### ZNF687 enhances HCC cell invasion and chemoresistance *in vitro*

**Figure 5:**
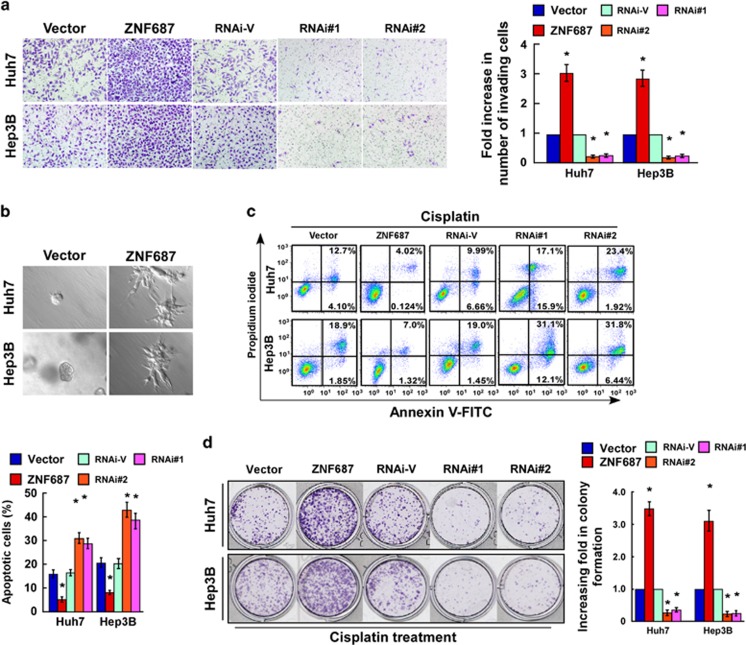
ZNF687 enhances HCC cell invasion and chemoresistance *in vitro.* (**a**) Representative images (left) and quantification (right) of invaded cells analyzed using Transwell assay. (**b**) Representative micrographs of cells grown on Matrigel for 10 days in 3D spheroid invasion assay. (**c**) Annexin V–fluorescein isothiocyanate/propidium iodide staining of cells treated with cisplatin (10 μm) for 24 h. Bars, mean±s.d. of three independent experiments. *P<0.05. (**d**) Representative images (left) and quantification (right) of colonies formed by cells treated with cisplatin (10 μM) for 2 weeks. Mean±s.d., *n*=3. *P<0.05.

CSCs are capable of self-renewal and have multi-lineage differentiation potential, which results in metastasis, chemotherapy resistance, and recurrence in HCC.^7, 8, 9, 10^ Consistent with TCGA data set GSEA findings that ZNF687 expression was significantly correlated with gene signatures related to HCC metastasis (Supplementary Figures 3A,B), *ZNF687* overexpression significantly increased HCC cell invasive capability, but *ZNF687* downregulation decreased it (Figure 5a). Furthermore, the three-dimensional (3D) spheroid invasion assay, considered a better mimic of *in vivo* tumor invasion, revealed that ZNF687-transduced HCC cell lines cultured in Matrigel for 10 days displayed morphologies typical of highly aggressive invasiveness, where there were more outward projections from nearly all individual cells, as opposed to the vector-transduced control cells (Figure 5b). These results suggest that elevated ZNF687 promotes HCC metastasis. Meanwhile, *ZNF687* overexpression conferred resistance to apoptosis in HCC cells treated with cisplatin, as indicated by higher colonogenic capability. However, silencing *ZNF687* significantly enhanced HCC cell sensitivity to cisplatin, resulting in fewer colonies (Figures 5c and d). These results indicate that ZNF687 has important roles in chemoresistance in HCC. GSEA confirmed these results in that ZNF687 expression was significantly correlated with gene signatures related to HCC metastasis and chemoresistance (Supplementary Figures 3A,B).

### ZNF687 contributed to HCC cell tumorigenic capability *in vivo*

**Figure 6:**
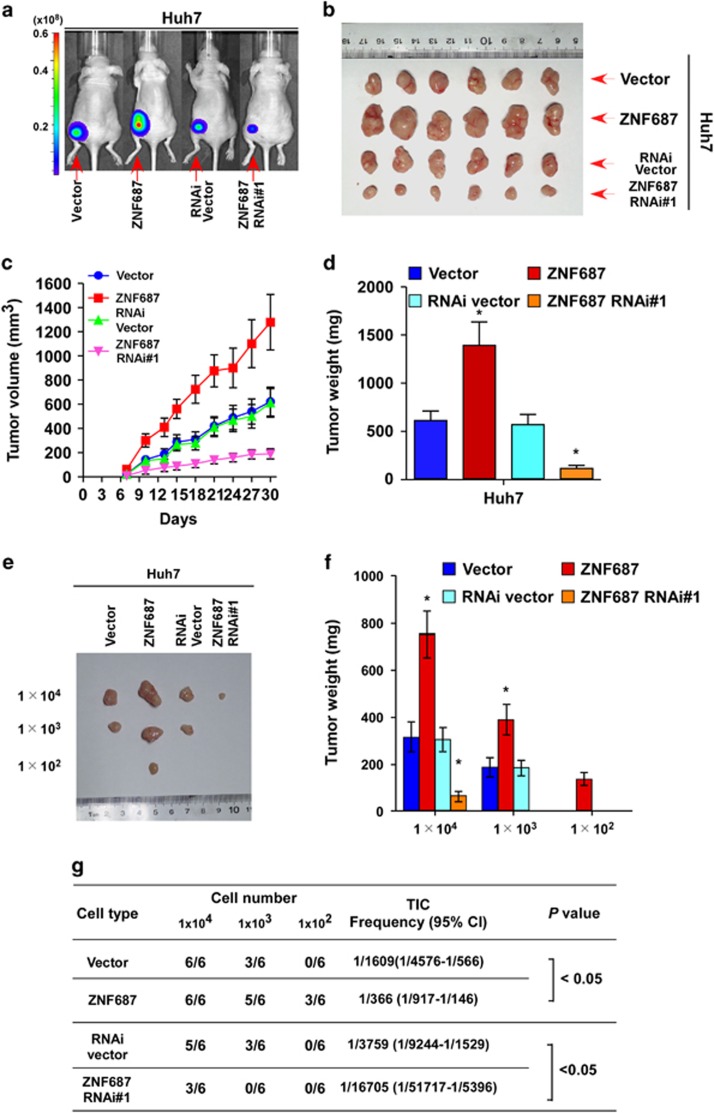
ZNF687 enhances tumorigenesis and stem cell-like traits of HCC *in vivo*. (**a**, **b**) Representative images of tumor-bearing mice (**a**) and tumors from all mice in each group (**b**). (**c**) Mean tumor volumes were measured on the indicated days. (**d**) Mean tumor weights. (**e**) Cells (1 × 10^4^, 1 × 10^3^ or 1 × 10^2^) were implanted into NOD/SCID mice. Representative images of tumorigenesis in each group are shown. (**f**) Histogram shows the mean tumor weights of each group (*n*=6 per group). *P<0.05. Mean tumor volumes are plotted. (**g**) Tumor formation rate of dilutions used and estimated percentage of tumor-initiating cells (TIC).

A xenograft tumor model was used to examine the biological effect of ZNF687 on HCC progression. Tumors formed by ZNF687-transduced Huh7 cells were larger and heavier than the tumors formed by the control cells (Figures 6a–d). Furthermore, to confirm the roles of ZNF687 in promoting HCC stem cell-like traits *in vivo*, we subcutaneously inoculated HCC cells mixed with Matrigel into nonobese diabetic/severe combined immunodeficient (NOD/SCID) mice. The weights and volumes of tumors formed by 1 × 10^4^, 1 × 10^3^ or 1 × 10^2^ ZNF687-transduced HCC cells were significantly greater than that formed by the control cells (Figures 6e–g; Supplementary Figure 4). Conversely, ZNF687-silenced HCC cells formed much smaller tumors and exhibited lower rates of tumorigenesis (Figures 6e–g; Supplementary Figure 4). Furthermore, extreme limiting dilution analysis (ELDA) revealed that ZNF687-transduced cells had greater tumorigenic capability, forming tumors when only 1 × 10^2^ cells had been implanted (Figure 6g). Taken together, these results demonstrate that ZNF687 strongly promotes HCC cell tumorigenicity *in vivo*.

### *ZNF687* directly targeted and activated *BMI1*, *NANOG* and *OCT4*

**Figure 7:**
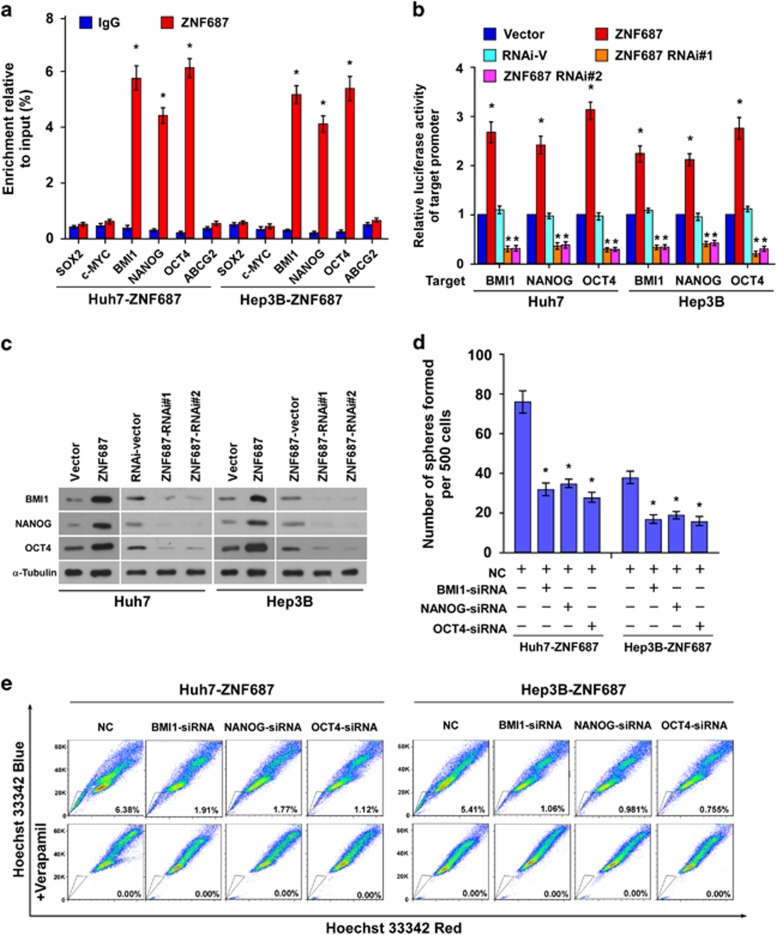
ZNF687 targets the *BMI1*, *NANOG* and *OCT4* promoters. (**a**) ChIP analysis of the physical association of regions of the target gene promoters with ZNF687. ChIP assays were performed using a FLAG antibody to identify ZNF687-binding sites in the target gene promoters. IgG, negative control. (**b**) Luciferase reporter gene assays of the target gene promoters. *Renilla* luciferase activity, normalized control. (**c**) Western blot analysis of BMI1, NANOG and OCT4 protein expression in Huh7 and Hep3B cells stably overexpressing ZNF687 or in which ZNF687 had been silenced. α-Tubulin, loading control. (**d**) Quantification of tumorspheres formed in ZNF687-overexpressing cells treated with BMI1, NANOG or OCT4 small interfering RNA (siRNA). (**e**) Hoechst 33342 dye exclusion was used to examine the effects of *BMI1*, *NANOG* or *OCT4* knockdown on the proportion of SP^+^ cells in ZNF687-overexpressing cells. Bars, mean±s.d. of three independent experiments. *P<0.05.

To explore the mechanism underlying the ZNF687-mediated stem cell-like traits of HCC cells, we predicted several possible downstream targets of ZNF687 using JASPAR database analysis. Chromatin immunoprecipitation–quantitative PCR (ChIP-qPCR) revealed that ZNF687 bound the *BMI1*, *NANOG* and *OCT4* promoters (Figure 7a). Furthermore, *ZNF687* overexpression markedly increased the luciferase reporter activity and protein expression of BMI1, NANOG and OCT4 in ZNF687-transduced cells, whereas *ZNF687* downregulation attenuated it (Figures 7b and c), demonstrating that *ZNF687* can directly target and activate the *BMI1*, *NANOG* and *OCT4* genes. Next, we investigated whether *BMI1*, *NANOG* or *OCT4* activation was required for the promoter effect of ZNF687 on the stem cell-like traits of HCC cells. Individually silencing *BMI1*, *NANOG* or *OCT4* in ZNF687-transduced cells (Supplementary Figure 5) potently reversed the enhanced tumorsphere forming ability and the increased proportion of SP^+^ cells in ZNF687-overexpressing HCC cells (Figures 7d and e). In short, these results demonstrate that ZNF687 promotes stem cell-like traits in HCC cells by directly targeting and activating *BMI1*, *NANOG* and *OCT4*.

### Clinical relevance between *ZNF687* and *BMI1*, *NANOG* and *OCT4* in HCC

**Figure 8:**
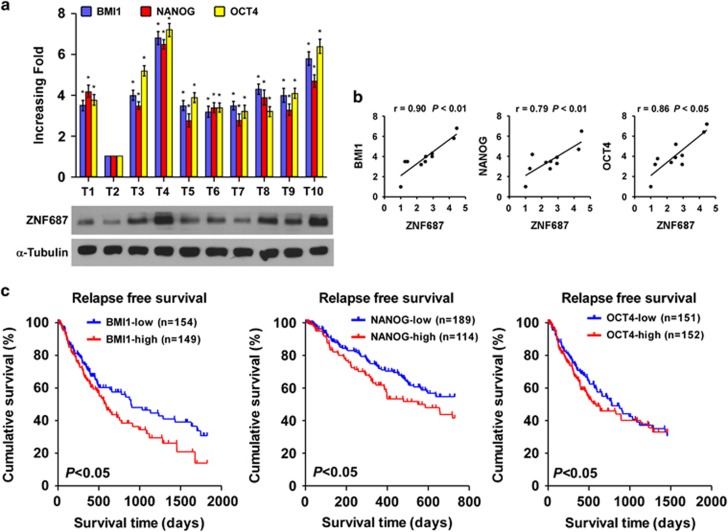
ZNF687 levels correlated with *BMI1*, *NANOG* and *OCT4* expression in HCC clinical tissues. (**a**) Expression analysis and (**b**) correlation analysis of ZNF687 expression and *BMI1*, *NANOG* and *OCT4* mRNA levels in 10 fresh HCC tissue samples (T). Bars, mean±s.d. of three independent experiments. (**c**) Kaplan–Meier analysis of relapse-free survival curves from TCGA data set of patients with HCC with relatively low or relatively high BMI1, NANOG and OCT4 expression (*P*<0.05). riments. *P<0.05.

We examined the clinical correlation between ZNF687 and the pluripotency-associated genes in human HCC tissues. Analysis of 10 freshly collected clinical HCC samples revealed that *ZNF687* mRNA expression was positively correlated with the mRNA expression of *BMI1* (*r*=0.90, *P*<0.01), *NANOG* (*r*=0.79, *P*<0.01) and *OCT4* (*r*=0.86, *P*<0.05) (Figures 8a and b). Kaplan–Meier analysis of TCGA data sets revealed that *BMI1*, *NANOG* and *OCT4* expression levels were significantly correlated with shorter relapse-free survival (*P*<0.05; Figure 8c), suggesting that BMI1, NANOG and OCT4 contribute to HCC recurrence. Collectively, these results further support the premise that *ZNF687* overexpression enhances HCC tumorigenic capability and promotes HCC recurrence by upregulating BMI1, NANOG and OCT4.

## Discussion

The key findings of the present study are that *ZNF687* overexpression contributes to CSC-like traits and promotes the tumor formation, invasion, and chemoresistance capabilities of HCC cells, and that ZNF687 can directly upregulate the transcription of the pluripotency-associated factors *BMI1*, *NANOG* and *OCT4*. Importantly, elevated *ZNF687* expression in human HCC tissues was closely correlated with poorer overall survival and relapse-free survival, indicating that ZNF687 might represent a valuable prognostic factor and potential target in HCC therapy.

Zinc finger proteins constitute an abundant family in the human genome, and regulate diverse biological processes, including cell development, differentiation, metabolism and autophagy.^24^ For example, ZNF687, a newly identified C2H2 ZNF, is highly expressed in the hematopoietic organs, such as the kidney and spleen, and is involved in regulating hematopoietic cell proliferation and differentiation.^21^ During caudal fin regeneration in zebrafish, *ZNF687* is greatly upregulated, and regulates osteoblast proliferation and differentiation.^21^ Furthermore, *ZNF687* mutations or translocation have been observed in AML and severe Paget disease of bone associated with GCT,^21^ suggesting that *ZNF687* dysregulation might contribute to cancer development and progression. Herein, we found that *ZNF687* expression was markedly upregulated at both mRNA and protein level in human HCC tissues, and that it correlated with poorer overall survival and relapse-free survival, suggesting that ZNF687 might be a potent oncogenic protein and involved in HCC progression.

Multiple independent studies have demonstrated that CSCs are involved regulating HCC tumorigenicity, metastasis, and chemoresistance, which results in recurrence and poor prognosis.^7, 10, 25, 26^ However, the critical factors that regulate CSC maintenance in HCC remain largely unknown. Recent studies have revealed that aberrant expression of zinc finger proteins contributes to progression in multiple cancers, including tumorigenicity, metastasis and chemoresistance,^27, 28, 29^ which the CSC subpopulation might regulate. Here, we found that *ZNF687* overexpression markedly promoted HCC cell tumorsphere formation capability and increased the HCC SP^+^/CD133^+^ populations *in vitro*. Furthermore, *ZNF687* overexpression promoted HCC cell tumorigenic capability *in vivo*. Hence, these results suggest that ZNF687 can participate in maintaining stem cell-like traits in HCC, which might represent a novel treatment target. On the other hand, CSCs also contribute to tumor metastasis, given their invasive and chemoresistance capacities,^30, 31, 32^ and correlate with cancer progression. Consistently, we also found that *ZNF687* overexpression enhanced HCC metastasis and chemoresistance, whereas *ZNF687* downregulation reduced it, and TCGA data set GSEA showed that high *ZNF687* expression correlated with the metastasis and chemoresistance signatures, further evidence that ZNF687 has crucial roles in HCC development and progression.

Numerous studies have demonstrated that multiple pluripotency-associated factors, such as *Oct4*, *Sox2*, *Klf4* (Kruppel-like factor 4), and c-*Myc*, can reprogram mouse fibroblasts into induced pluripotent stem cells and promote cancer progression.^33, 34^ Despite a cohort of studies on the pluripotency-associated factor regulation of CSC population maintenance, the regulatory mechanisms differ. We found that ZNF687 significantly upregulated the expression of the pluripotency-associated factors, including *SOX2*, c-*MYC*, *BMI1*, *NANOG*, *OCT4* and *ABCG2*. ZNF687 physically interacts with the MBD3/NuRD (methyl-CpG–binding domain protein 3/nucleosome remodeling and deacetylases) complex,^35^ which is an active enhancer and can facilitate the induction of pluripotency with NANOG,^36^ suggesting that ZNF687 might activate transcription and be involved in the regulation of cell reprogramming. ZNF687 can also interact with ZMYND8 (zinc finger MYND-type containing 8) and ZNF592 to form a Z3 transcriptional coregulator complex,^37^ which is associated with the H3K4 demethylation machinery, indicating that ZNF687 might have a prominent role in chromatin remodeling for transcription. In the present study, we found that ZNF687 could directly target and activate *BMI1*, *NANOG* and *OCT4* in HCC cells, whereas individually silencing *BMI1*, *NANOG* or *OCT4* potently reduced tumorsphere formation and the SP^+^ subpopulation in ZNF687-overexpressing HCC cells. Collectively, ZNF687 could induce stem cell-like traits in HCC cells via transcriptional regulation of the pluripotency-associated factors, suggesting that ZNF687 might be involved in regulating HCC progression.

SOX2, c-MYC and ABCG2 are upregulated transcriptionally by the Wnt/β-catenin signaling pathway,^38, 39, 40^ which is aberrantly activated in HCC and has important roles in CSC maintenance.^41, 42, 43^ Interestingly, we found that ZNF687 overexpression significantly increased the luciferase activity of the TOPflash/FOPflash reporter but that ZNF687 downregulation decreased it, suggesting that ZNF687 may be involved in the activation of Wnt/β-catenin signaling (Supplementary Figure 6A). Importantly, Wnt/β-catenin signaling inhibition by ICG-001, a specific Wnt/β-catenin signaling inhibitor, greatly decreased SOX2, c-MYC, and ABCG2 expression in ZNF687-transduced HCC cells (Supplementary Figure 6B). These results suggest that Wnt/β-catenin signaling might contribute to ZNF687-induced upregulation of SOX2, c-MYC and ABCG2. Therefore, further investigation of the mechanism by which ZNF687 activates Wnt/β-catenin signaling is worthwhile.

## Materials and methods

### Cells and cell culture

Primary cultures of normal human hepatocytes (Normal 1 and Normal 2) were established from fresh specimens of adjacent non-tumor hepatocellular tissue according to a previously reported protocol.^44^ The human HCC cell lines QGY-7701, PLC/PRF/5, Huh7, BEL-7404, Hep3B, MHCC97H, HepG2 and QGY-7721 were grown in Dulbecco’s modified Eagle’s medium (DMEM; Invitrogen, Carlsbad, CA, USA) supplemented with 10% fetal bovine serum (HyClone, Logan, UT, USA). The cell lines were routinely monitored by microscopic morphology examination and were routinely treated with commercial ciprofloxacin (Bayer, Leverkusen, Germany) according to the manufacturer’s instructions. Mycoplasma eradication was evaluated by PCR.

### Patient information and tissue specimens

A total 204 paraffin-embedded archived HCC samples were histopathologically and clinically diagnosed at the Third Affiliated Hospital of Sun Yat-Sen University. We obtained prior patient consent and institutional research ethics committee approval for the use of these clinical materials for research purposes. The clinical information of the patient samples is summarized in Supplementary Table 1. Ten fresh HCC specimens and the paired adjacent non-cancerous tissues were frozen and stored in liquid nitrogen until further use.

### Vectors, retroviral infection and transfection

A ZNF687 expression construct was generated from PCR-amplified complementary DNA (cDNA) and cloned into pMSCV plasmid, and shRNA oligonucleotide sequences targeting ZNF687 were cloned into pSuper.retro.puro viral vector. The respective promoters of the pluripotency-associated factors, for example, *BMI1*, *NANOG* and *OCT4*, spanning nucleotides −2000 to +500 (relative to the transcription initiation site), were cloned into pGL3 luciferase reporter plasmid (Promega, Madison, WI, USA). Plasmid transfection was performed using Lipofectamine 3000 (Invitrogen) according to the manufacturer’s instruction. Retroviral production and infection were performed using a previously reported protocol.^45^ Stable cell lines expressing ZNF687 or ZNF687-shRNA(s) were selected for 10 days with 0.5 μg/ml puromycin 48 h after infection.

### Western blot analysis

Western blotting was performed using antibodies against ZNF687 (1:500; Abcam, Cambridge, MA, USA), BMI1 (Cell Signaling Technology, Danvers, MA, USA), OCT4, and NANOG (Abcam). The blotting membranes were stripped and re-probed with anti–α-tubulin antibody (Sigma, St. Louis, MO, USA) as the loading control.

### Luciferase reporter assay

Cells (1 × 10^4^) were seeded in triplicate in 48-well plates and allowed to settle for 24 h. Luciferase reporter plasmids (100 ng) or control luciferase plasmid (100 ng) plus 5 ng pRL-TK *Renilla* plasmid (Promega) were transfected into HCC cells using Lipofectamine 3000 (Invitrogen). Luciferase and *Renilla* signals were measured 48 h after transfection using a Dual Luciferase Reporter Assay (Promega) according to the manufacturer’s instructions. Three independent experiments were performed, and the data are presented as the mean±s.d.

### Chromatin immunoprecipitation

ZNF687-overexpressing cells (2 × 10^6^) were treated with 1% formaldehyde to cross-link proteins to DNA in a 100-mm culture dish. Cell lysates were collected in a 15-ml tube and sonicated to shear the DNA into 300–1000-bp fragments. Aliquots containing equal amounts of chromatin supernatants were incubated with 1 μg anti-FLAG (Abcam) or anti–immunoglobulin G (IgG) antibody (Millipore) overnight at 4 °C with rotation. Following reverse cross-linking of protein/DNA complexes to free the DNA, real-time PCR was performed.

### Tumorsphere formation assay

Five hundred cells were seeded in 6-well ultra-low cluster plates (Corning, NY, USA) for 10 days. Spheres were cultured in DMEM/F12 serum-free medium (Invitrogen, Grand Island, NY, USA) supplemented with 2% B27 (Invitrogen, Grand Island, NY, USA), 20 ng/ml epidermal growth factor, 20 ng/ml basic fibroblast growth factor (PeproTech, Offenbach, Germany), 5 μg/ml insulin and 0.4% bovine serum albumin (Sigma).

### Tumor xenografts

The Sun Yat-Sen University Institutional Animal Care and Use Committee approved all experimental procedures. One group of NOD/SCID mice (6–7 weeks old; 18–20 g) were randomly divided into four groups (*n*=6 per group). All mice were inoculated subcutaneously with 1 × 10^6^ Huh7 cells in the left dorsal flank. Tumors were examined every 3 days; tumor length and width were measured using calipers; tumor volumes were calculated using the equation (*L* × *W*^2^)/2. On day 30, tumors were detected using an IVIS imaging system, and then the animals were euthanized, and the tumors were excised. Another group of NOD/SCID mice (6–7 weeks old; 18–20 g) were randomly divided into three groups (*n*=6 per group). Huh7 cells (1 × 10^4^, 1 × 10^3^ and 1 × 10^2^) were inoculated subcutaneously together with Matrigel (final concentration, 25%) into the inguinal folds of the mice. Tumor volume was determined using an external caliper and was calculated as above. The mice were sacrificed 60 days after inoculation, and the tumors were excised and subjected to pathologic examination.

### Microarray data processing and visualization

Microarray data were downloaded from TCGA data sets (http://cancergenome.nih.gov/) and the Gene Expression Omnibus database (http://www.ncbi.nlm.nih.gov/geo/) using the indicated accession numbers. GSEA was performed using GSEA 2.0.9 (http://www.broadinstitute.org/gsea/).

### Statistical analysis

All statistical analyses were performed using SPSS 16.0 (IBM, Armonk, NY, USA). Pearson’s chi-square test was used to study the correlation between *ZNF687* expression and the HCC clinicopathological characteristics. The survival curves of high- and low-ZNF687 expression patients were plotted using the Kaplan–Meier method; statistical differences were compared using a log-rank test. Univariate and multivariate statistical analyses were performed using a Cox regression model. Groups were compared using Student’s *t*-test. Data are the mean±s.d. Bivariate correlations between variables were calculated using Pearson’s rank correlation coefficients. *P*<0.05 was considered statistically significant.
