# Supplementary material for: Overexpression of zinc finger protein 687 enhances tumorigenic capability and promotes recurrence of hepatocellular carcinoma
Source: Oncogenesis. 2017 Jul 24;6(7):e363–. doi: 10.1038/oncsis.2017.63 (PMC5541715; doi:10.1038/oncsis.2017.63)
Supplement: Supplementary Information [file oncsis201763x1.docx]

**Supplemental Information**

**Supplemental Methods**

**Primers and Oligonucleotides**

Cloning primer human ZNF687-ORF, forward:5’-GCCGGATCCGCCATGGGGGATATG AAGACCCCTGA-3’ and reverse 5’-GCCGAATTCCTAGTTGTCCCCAACAGCCC -3’; Real-time PCR primer:ZNF687, forward: 5’-CCCCAAAGTGCCCGTCTGTC-3’ and reverse: 5’-TGTGATATTCCCGCAGGATGTTTT-3’; SOX2 forward:5’-GCTTAGCCTCG TCGATGAAC-3’; and reverse: 5’-AACCCCAAGATGCACAACTC-3’; c-MYC forward: 5’-CACCGAGTCGTAGTCGAGGT-3’; and reverse: 5’-GCTGCTTAGACGCTGGATTT- 3’; BMI1 forward: 5’-TCGTTGTTCGATGCATTTC T-3’; and reverse: 5’-CTTTCATTGT CTTTTCCGCC-3’; OCT4 forward: 5’-GGGTTCTCGATACTGGTTCGC-3’; and reverse: 5’-GTGGAGGAAGCTGACAACAA-3; NANOG forward: 5’-ATGGAGGAGGGAAGAG GAGA-3’; and reverse: 5’-GATTTGTGGGC CTGAAGAAA -3’; ABCG2 forward: 5’-TG GTGTTTCCTTGTGACACTG-3’; and reverse: 5’- TGAGCCTTTGGTTAAGACCG-3’; GAPDH forward: 5’-AATGAAGGGGTCATTGATGG-3’; and reverse: 5’-AAGGTGAAG GTCGGAGTCAA -3’. The housekeeping gene GAPDH was used as internal controls for mRNAs. Expression levels of genes were calculated as 2-[(Ct of genes) - (Ct of GAPDH)]. (Ct represents the threshold cycle for each transcript). ZNF687 RNAi#1: GCGCATGCAT AAGAATCGA; ZNF687 RNAi#2: CTCTTAAGGTGCGGATCAA；BMI1 RNAi: GAGATA ATAAGCTTGTCTA; NANOG RNAi: GCATGCAGTTCCAGCCAAA; OCT4 RNAi: GCT TCAAGAACATGTGTAA.

**RNA extraction, reverse transcription and real-time PCR**

Total RNA from cultured cell and surgically obtained tumor tissues was extracted using the Trizol (Life Technologies) reagent according to the manufacturer’s instruction. Real-time reverse transcription-polymerase chain reaction (PCR) primers and probes were designed with the assistance of the Primer Express v 2.0 software (Applied BioSystems, Foster, CA). Expression data were normalized to the geometric mean of housekeeping gene *GAPDH* to control the variability in expression levels and calculated as 2^-[(^*^Ct^* ^of gene) – (^*^Ct^* ^of^ *^GAPDH^*^)]^, where Ct represents the threshold cycle for each transcript.

**Immunohistochemistry (IHC)**

Hepatocellular carcinoma tissues were analyzed using immunohistochemistry (IHC) analysis with anti-ZNF687 antibody (Sigma, Saint Louis, MO). The degree of immunostaining for protein expression in tumor and normal tissues was quantitatively analyzed by using the AxioVision 4.6 computerized image analysis system assisted with an automatic measurement

program (Carl Zeiss). The method of mean optical density (MOD) was used to determine the immunostaining of each tested specimen. Briefly, the stained slides were evaluated at 200x magnification using the SAMBA 4000 computerized image analysis system with Immuno 4.0 quantitative program (Image Products International, Chantilly, Virginia), and ten random picked random fields in each specimen were analyzed to determine the MOD of the specimen, based on which the mean MOD of a study group of samples was further generated for subsequent inter-group comparative analysis. A negative control with each batch of staining was used for background subtraction in the quantitative analysis. In order to avoid possible bias in selecting fields that are more cellular or heavily stained, the representative staining fields of each tumor sample were analyzed and scored independently by two observers. The MOD data were statistically analyzed by using the *t* test to compare the average MOD difference between different groups of tissues, which MOD ≥ average MOD to define tumors as high expression, and MOD < average MOD to define tumors as low expression, and *P* < 0.05 was considered significant.

**Flow cytometric analysis**

HCC cells were digested with trypsin and re-suspended at 1 × 10^6^ cells per ml in DMEM that contained 2% fetal bovine serum (FBS) and then were pre-incubated at 37°C for 30 min with or without 100 μM verapamil (Sigma-Aldrich, Germany) to inhibit ABC transporters. The cells were subsequently incubated for 90 min at 37°C with 5 μg/ml Hoechst 33342 (Sigma-Aldrich, Germany). Finally, the cells were incubated on ice for 10 min and washed with ice-cold PBS before flow cytometry analysis. The data were analyzed using Summit 5.2 software (Beckman Coulter, Indianapolis, IN). CD133 expression studies were performed according to the instructions in a Miltenyi Biotec kit (Bergisch Gladbach, Germany). Briefly, freshly sorted cells were blocked using a FcR Blocking Reagent kit (Miltenyi Biotec) and labeled with fluorescein isothiocyanate–conjugated anti-human CD133/2 (Miltenyi Biotec). Isotype-matched mouse antibodies (Miltenyi Biotec) served as controls. Flow cytometry analysis was performed using a Gallios flow cytometer (Beckman Coulter, Brea, CA, USA) and the data were analyzed using FlowJo 7.6 software (TreeStar Inc., Ashland, OR, USA).

**Transwell assay.** Cells (2×10^4^) were analyzed using the Transwell chambers assay (Costar; Corning Inc.). The lower chamber of the Transwell device was filled with 500 μl DMEM supplemented with 10% FBS. After 24 hours of incubation, cells invading into the bottom side of the inserts were fixed in 1% paraformaldehyde, stained with hematoxylin, and counted (Ten random 100×fields per well). Cell counts were expressed as the mean number of cells per field of view. Three independent experiments were performed and the data are presented as mean ± standard deviation (SD).

**3D spheroid invasion assay.** Indicated cells (3×10^5^) were trypsinized and seeded on 2% Matrigel coated in 24-well plates, and medium was refreshed every other day. Cells forming a 3D spherical structure (spheres) were photographed at 2-day intervals for 10 days.

**Cell Clonogenic Survival Assay.** Indicated Cells were plated in 6-well plates (5×10^3^ cells per plate) in normal condition for overnight. The cells were treated with 5μM cisplatin for 2 weeks. Culture medium was changed once a week. and cultured for 10 days. After 2-week incubation, the colonies were washed with 1×PBS and in 4% paraformaldehyde . The colonies were stained with 1% crystal violet for 20 mins. The number of colonies (defined as cell clusters consisting of at least 50 cells) was quantified by Analysis software (Olympus Biosystems).

**Annexin V Assay.** The ApopNexinTM FITC Apoptosis Detection Kit (Millipore, Lake Placid, NY) was used for quantification of apoptotic cells, according to manufacturer’s instruction. Indicated treated cells were washed with PBS and the Annexin-V binding solution, subsequently added 150 μl of an Annexin-V antibody in Binding Buffer and incubated for 15 min, followed by addition of 1.5 μl of PI at 1 mg/ml and a further incubation for 5 min. After washing with the Annexin-V Binding Buffer, positive Annexin-V staining was visualized under a fluorescence microscope equipped with a filter for fluorescein isothiocyanate (excitation: 490 nm, emission: 525 nm), and PI staining was assessed with the filter for Texas red (excitation: 570 nm, emission: 610 nm).

**Cell Treatments.** Cisplatin (5 μM; Selleck Chemicals, Houston, TX) or β-catenin Inhibitor ICG-001 (10μM, Selleck Chemicals, Houston, TX) were dissolved in dimethyl sulfoxide and incubated indicated cells for 24 hours.

**SUPPLEMENTARY FIGURE LEGENDS**

**Supplementary Figure S1.** *ZNF687* mRNA is overexpressed in HCC cell lines and primary human HCC tissue. (**A, B**) Real-time PCR analysis of *ZNF687* expression in two primary normal liver cell lines and eight cultured HCC cell lines (**A**) and in ten primary HCC tissues (T) and matched adjacent non-tumor tissues (ANT) (**B**). Transcript levels were normalized by *GAPDH* expression. Bars, mean ± SD of three independent experiments.**P* < 0.05.

**Supplementary Figure S2.** Correlation between *ZNF687* expression and CSC gene signatures. GSEA plot showing that high *ZNF687* expression correlated positively with stem cell gene signatures in published TCGA expression profiles of patients with HCC.

**Supplementary Figure S3.** Correlation between *ZNF687* expression and metastasis and chemoresistance gene signatures. GSEA plot showing that high *ZNF687* expression correlated positively with metastasis and chemoresistance gene signatures in published TCGA expression profiles of patients with HCC.

**Supplementary Figure S4.** ZNF687 enhances stem cell–like traits of HCC *in vivo*. Growth curves of tumor formation after implantation 1 × 10^4^ (left), 1 × 10^3^ (middle), or 1 × 10^2^ (right) HCC cells.

**Supplementary Figure S5.** *BMI1*, *NANOG*, and *OCT4* silencing. Western blot analysis of BMI1, NANOG, and OCT4 in HCC cells transfected with BMI1, NANOG, and OCT4 siRNA. NC, negative control.

**Supplementary Figure S6.** ZNF687 might induce SOX2, c-MYC, and ABCG2 expression via Wnt/β-catenin signaling. (**A**) TOPflash/FOPflash luciferase activity. (**B**) Real-time PCR analysis of *SOX2*, c-*MYC*, and *ABCG2* expression in HCC cell lines treated with vehicle or ICG-001 (10 μM). Transcript levels were normalized by *GAPDH* expression. Bars, mean ± SD of three independent expe**Supplementary Tables**

**Supplementary Table 1**. Clinicopathological characteristics and ZNF687 expression in 204 patients with HCC.

| **Clinical features** | **Number** | | **(%)** |
| --- | --- | --- | --- |
| **Gender** |  | |  |
| Male | 170 | | 83.3 |
| Female | 34 | | 16.7 |
| **Age (years)** |  | |  |
| ≤60 | 167 | | 81.8 |
| >60 | 37 | | 19.2 |
| **Clinical stage** |  | |  |
| I/II | 89 | | 43.6 |
| III/IV | 115 | | 56.4 |
| **Histological differentiation**  Well/Moderate  Poor | | 99  105 | 48.5  51.5 |
| **T classification** |  | |  |
| T_1_/T_2_ | 88 | | 43.1 |
| T_3_/T_4_ | 116 | | 56.9 |
| **N classification** |  | |  |
| N_0_ | 178 | | 87.2 |
| N_1_ | 26 | | 12.8 |
| **M classification** |  | |  |
| No | 160 | | 78.4 |
| Yes | 44 | | 21.6 |
| **HBsAg** |  | |  |
| Negative | 20 | | 9.8 |
| Positive | 184 | | 91.2 |
| **AFP** |  | |  |
| > 400**ng/ml** | 73 | | 35.7 |
| ≤ 400**ng/ml** | 131 | | 64.3 |
| **Vital status** |  | |  |
| Alive | 109 | | 53.4 |
| Dead | 95 | | 46.6 |
| **Expression of ZNF687** |  | |  |
| Low expression | 90 | | 44.1 |
| High expression | 114 | | 55.9 |

**Supplementary Table 2**. Correlation between ZNF687 expression with clinicopathological features in patients with HCC.

| **Clinical feateres** | | **ZNF687 expression** | | ***P*-value** |
| --- | --- | --- | --- | --- |
|  |  | **Low** | **High** |  |
| **Gender** | Male | 74 | 96 | 0.71 |
|  | Female | 16 | 18 |  |
| **Age (years)** | ≤60 | 78 | 89 | 0.143 |
|  | >60 | 12 | 25 |  |
| **Clinical stage** | I/II | 56  132  62 | 33  44 | <0.001 |
|  | III/IV | 34 | 81 |  |
| **Histological**  **differentiation** | Well/Moderate  Poor | 64  26 | 35  79 | <0.001 |
| **T classification** | T_1_/ T_2_ | 55  64  106 | 33  18 | <0.001 |
|  | T_3_/ T_4_ | 35 | 81 |  |
| **N classification** | N_0_ | 84  204 | 94  0 | 0.021 |
|  | N_1_ | 6 | 20 |  |
| **M classification** | No | 77  204 | 83  0 | 0.039 |
|  | Yes | 13 | 31 |  |
| **HBsAg** | Negative | 8  174 | 12 | 0.814 |
|  | Positive | 82 | 102 |  |
| **AFP** | > 400ng/ml | 30 | 43 | 0.558 |
|  | ≤ 400ng/ml | 60 | 71 |  |

**Supplementary Table 3**. Univariate and multivariate analysis of different prognostic parameters in patients with HCC by Cox-regression analysis.

|  | **Univariate analysis** | | **Multivariate analysis** | |
| --- | --- | --- | --- | --- |
|  | ***P*** | **Hazard ratio**  **(95% CI)** | ***P*** | **Hazard ratio**  **(95% CI)** |
| **Clinical stage** | <0.001 | .995  (1.549-2.569) | <0.001 | 1.7706  (1.277-2.281) |
| **T classification** | <0.001 | 1.681  (1.307-2.161) | 0.112 | 1.259  (0.948-1.672) |
| **N classification** | 0.027 | 1.746  (1.066-2.862) | 0.667 | 1.128  (0.652-1.951) |
| **M classification** | 0.016 | 1.696  (1.105-2.604) | 0.594 | 0.870  (0.521-1.452) |
| **ZNF687 expression** | <0.001 | 2.449  (1.598-3.751) | 0.013 | 1.813  (1.133-2.900) |
