# Supplementary figures and images for: Overexpression of zinc finger protein 687 enhances tumorigenic capability and promotes recurrence of hepatocellular carcinoma
Source: Oncogenesis. 2017 Jul 24;6(7):e363–. doi: 10.1038/oncsis.2017.63 (PMC5541715; doi:10.1038/oncsis.2017.63)

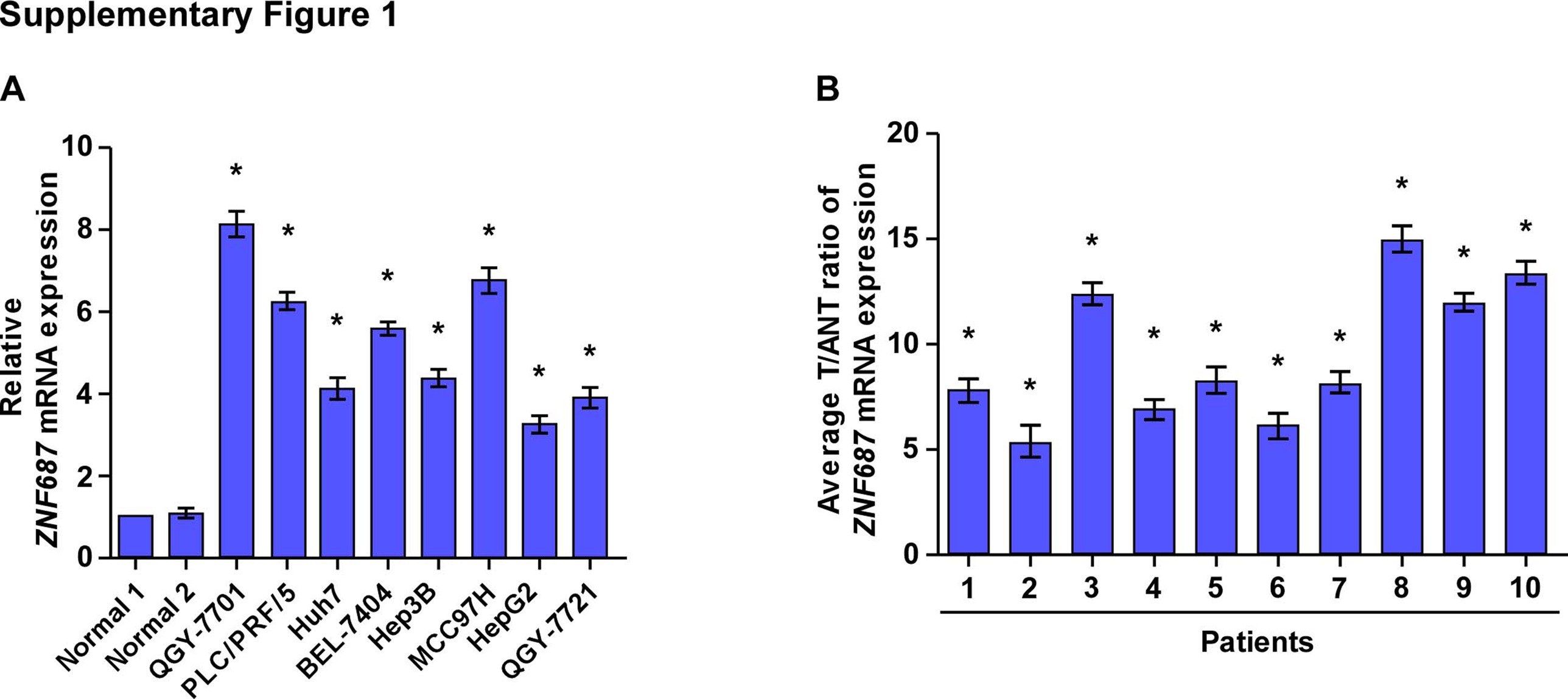

Supplement: Supplementary Figure 1 [file oncsis201763x2.tif]

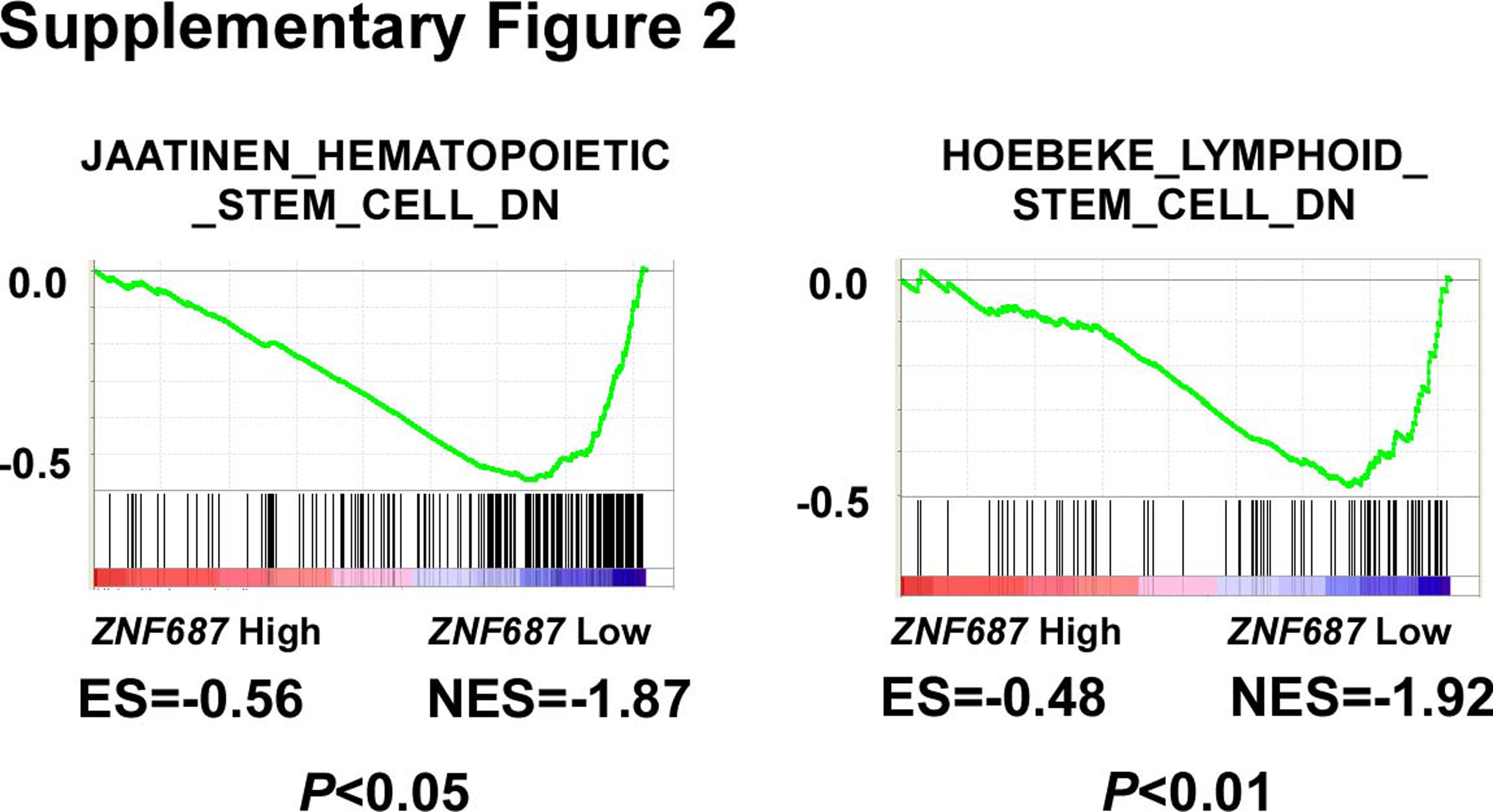

Supplement: Supplementary Figure 2 [file oncsis201763x3.tif]

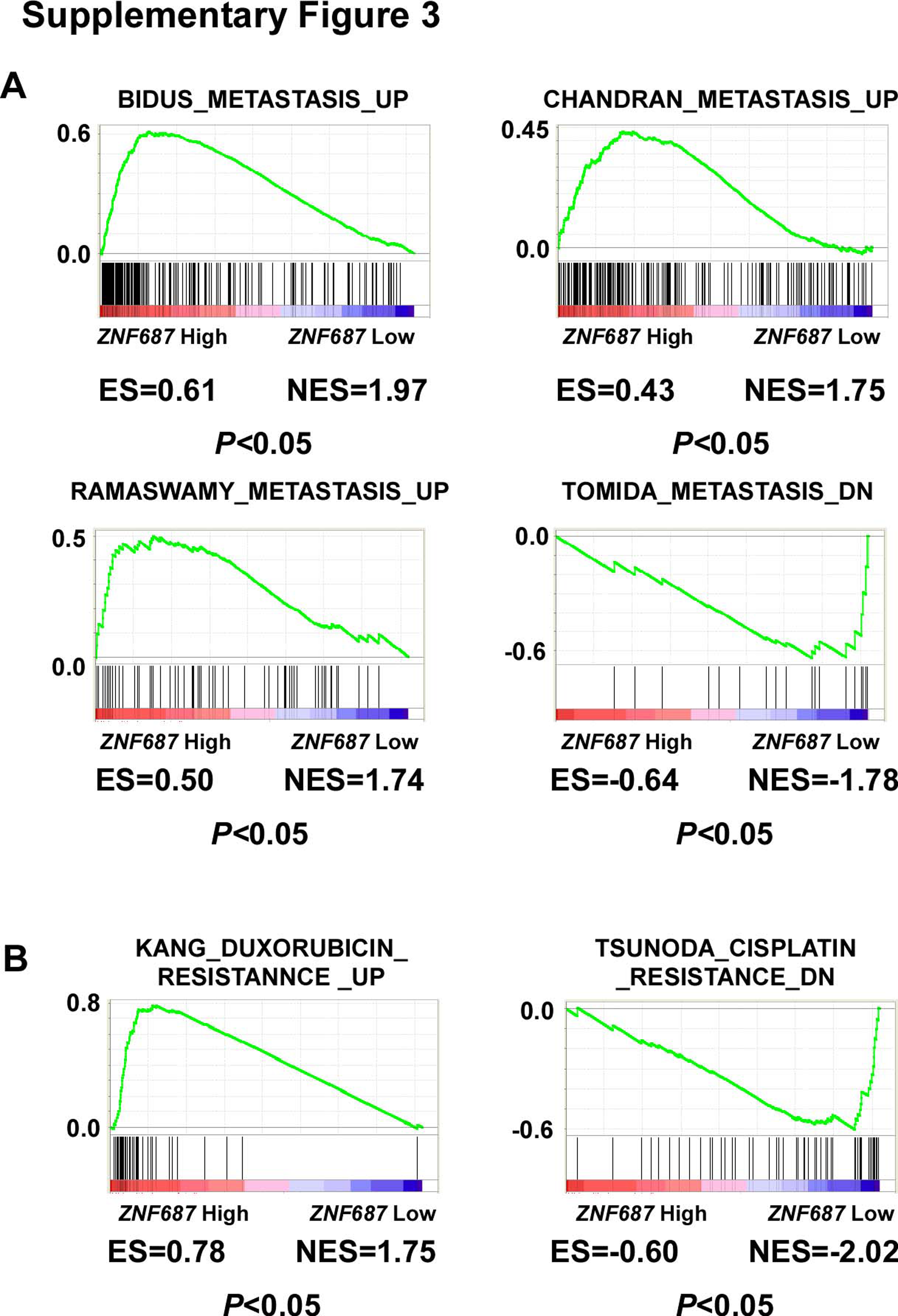

Supplement: Supplementary Figure 3 [file oncsis201763x4.tif]

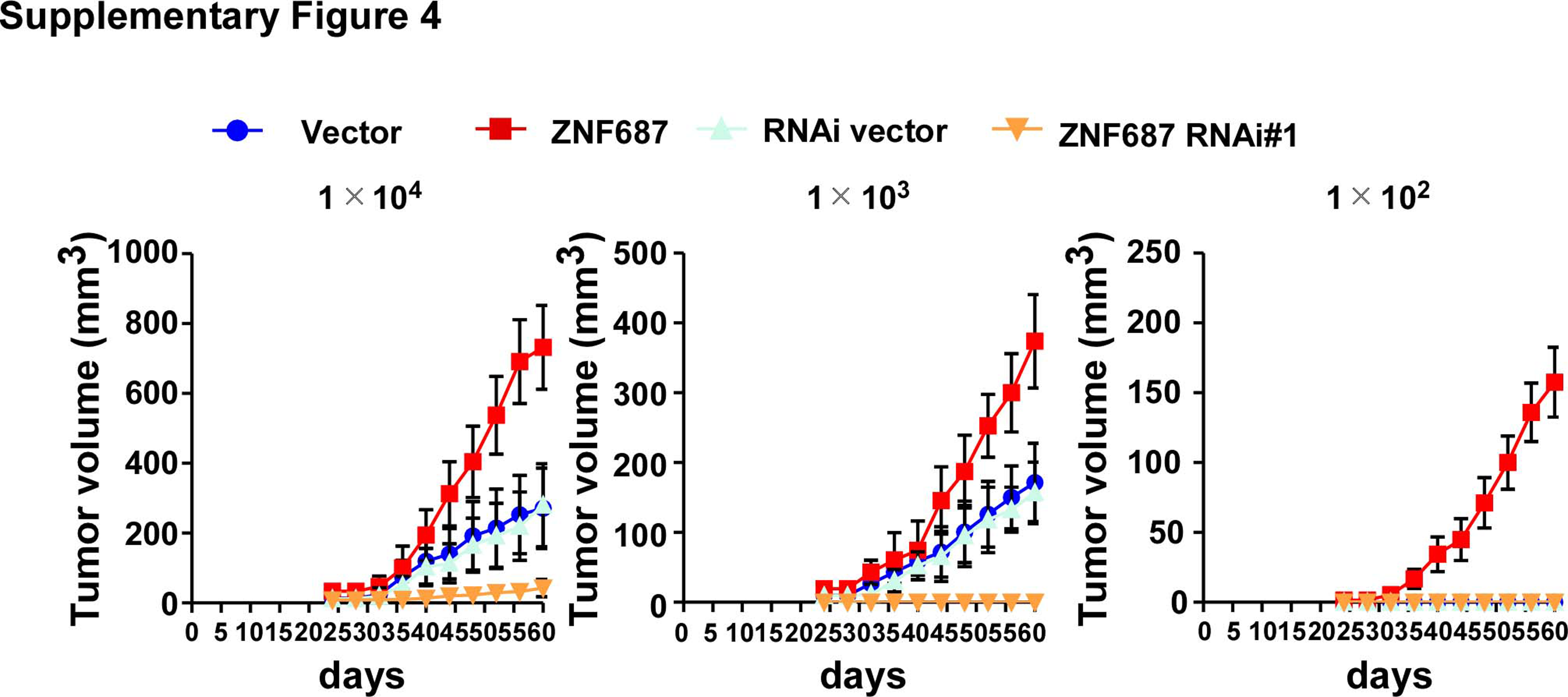

Supplement: Supplementary Figure 4 [file oncsis201763x5.tif]

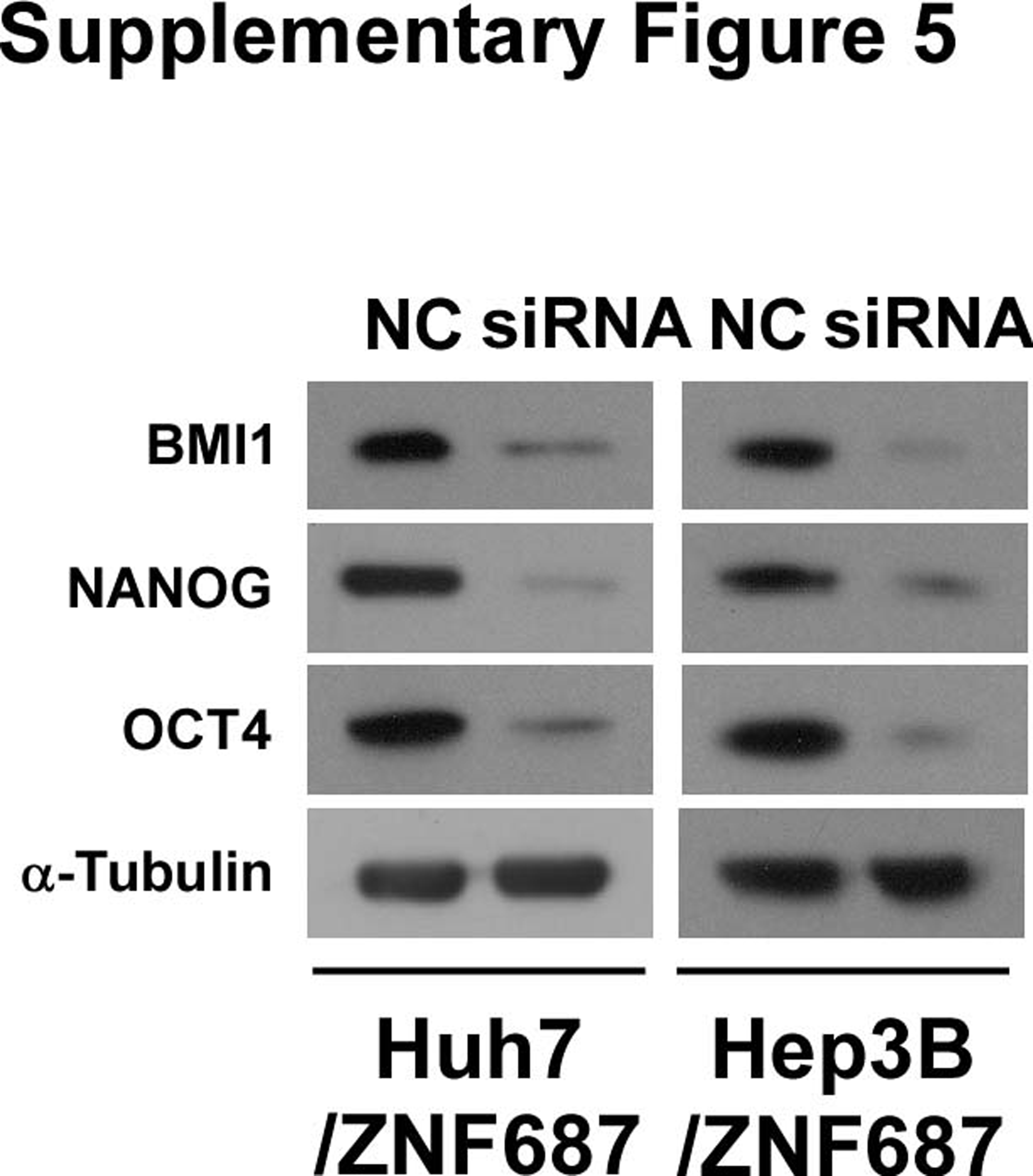

Supplement: Supplementary Figure 5 [file oncsis201763x6.tif]

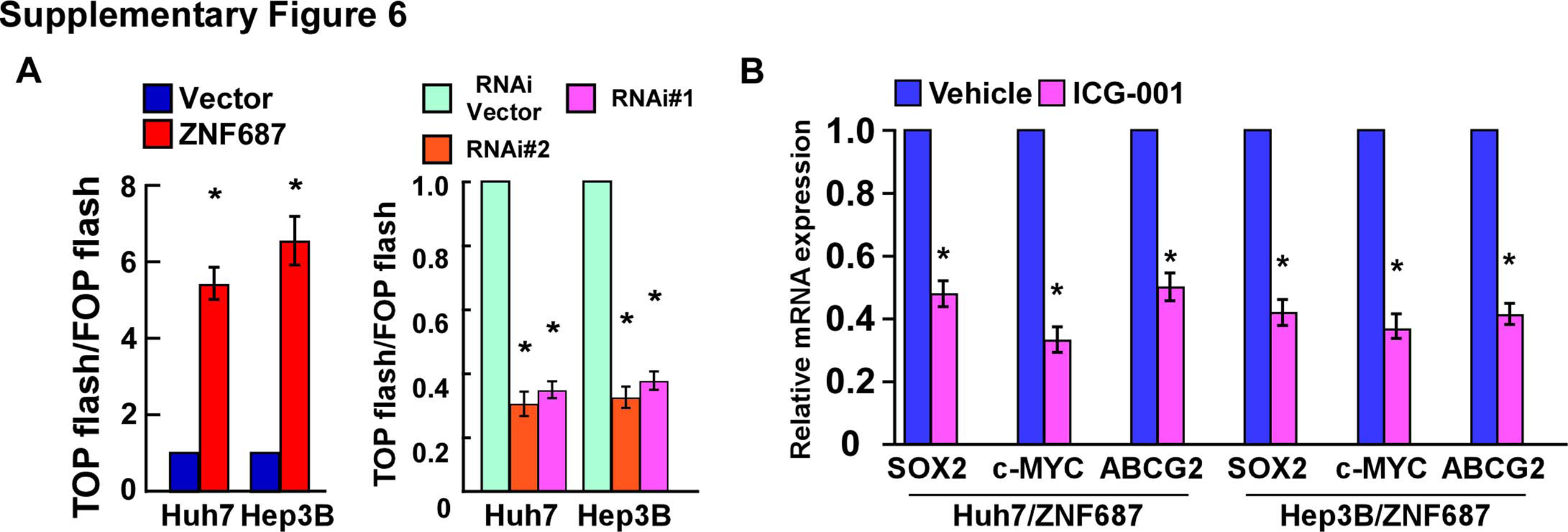

Supplement: Supplementary Figure 6 [file oncsis201763x7.tif]
